# Genetic, Cytogenetic and Morphological Trends in the Evolution of the *Rhodnius* (Triatominae: Rhodniini) Trans-Andean Group

**DOI:** 10.1371/journal.pone.0087493

**Published:** 2014-02-03

**Authors:** Sebastián Díaz, Francisco Panzera, Nicolás Jaramillo-O, Ruben Pérez, Rosina Fernández, Gustavo Vallejo, Azael Saldaña, Jose E. Calzada, Omar Triana, Andrés Gómez-Palacio

**Affiliations:** 1 Grupo BCEI, Universidad de Antioquia UdeA, Medellin, Antioquia, Colombia; 2 Sección Genética Evolutiva, Facultad de Ciencias, Universidad de la República, Montevideo, Montevideo, Uruguay; 3 Laboratorio de Investigación en Parasitología Tropical, Universidad del Tolima, Ibagué, Tolima, Colombia; 4 Instituto Conmemorativo Gorgas de Estudios de la Salud (ICGES), Ciudad de Panamá, Panamá, Panamá; Universidade Federal do Rio de Janeiro, Brazil

## Abstract

The *Rhodnius* Pacific group is composed of three species: *Rhodnius pallescens*, *R. colombiensis* and *R. ecuadoriensis,* which are considered important vectors of trypanosomes (*Trypanosoma cruzi* and *T. rangeli*) infecting humans. This group is considered as a recent *trans-Andean* lineage derived from the widespread distributed sister taxa *R. pictipes* during the later uplift of northern Andes mountain range. The widest spread species *R. pallescens* may be a complex of two divergent lineages with different chromosomal attributes and a particular biogeographical distribution across Central America and Colombia with several southern populations in Colombia occupying the same sylvatic habitat as its sister species *R. colombiensis.* Although the taxonomy of *Rhodnius* Pacific group has been well studied, the unresolved phylogenetic and systematic issues are the target of this paper. Here we explore the molecular phylogeography of this species group analyzing two mitochondrial (ND4 and cyt b) and one nuclear (D2 region of ribosomal 28S gene) gene sequences. The molecular analyses suggest an early divergence of the species *R. ecuadoriensis* and *R. colombiensis,* followed by a recent expansion of *R. pallescens* lineages. The phylogenetic relationship between sympatric *R. pallescens* Colombian lineage and *R. colombiensis* was further explored using wing morphometry, DNA genome size measurements, and by analyzing chromosomal behavior of hybrids progeny obtained from experimental crosses. Our results suggest that the diversification of the two *R. pallescens* lineages was mainly influenced by biogeographical events such as (i) the emergence of the Panama Isthmus, while the origin and divergence of *R. colombiensis* was associated with (ii) the development of particular genetic and chromosomal features that act as isolation mechanisms from its sister species *R. pallescens* (Colombian lineage). These findings provide new insights into the evolution of the *Rhodnius* Pacific group and the underlying biological processes that occurred during its divergence.

## Introduction

The subfamily Triatominae (Hemiptera: Reduviidae) includes 144 species of hematophagous insects classified in 5 tribes and 15 genera [1]–[5]. They are vectors of the protozoan parasite *Trypanosoma cruzi,* causative agent of American Trypanosomiasis or Chagas disease, one of the most important parasitic diseases in Latin America [6].

One prominent Triatominae tribe, the Rhodniini, is comprised of two genera: *Rhodnius* (18 species) and *Psammolestes* (three species) [2]–[5], a few species of which are targets of several vector control initiatives in Andean and Central American countries [6]. Within this tribe are the pictipes and robustus lineages that are recognized by molecular, biochemical, morphometric and biogeographical attributes [7], [8]. The pictipes lineage gave rise to the *trans-Andean* (pallescens) and Amazonian (pictipes) species groups, while the robustus lineage diversified within Amazonia and spread to neighbouring ecoregions (Orinoco, Cerrado-Caatinga-Chaco, and Atlantic Forest) [9]. The pallescens group, composed of *R. pallescens, R. colombiensis* and *R. ecuadoriensis*, is also named the *trans-Andean* or Pacific group because its geographic distribution is basically restricted to coastal regions of Ecuador, Colombia, Panamá and Costa Rica. *Rhodnius ecuadoriensis* is the most southerly-distributed species of the Pacific group and is restricted to southern Ecuador and northern Peru. This species occupies palms trees (*Phytelephas aequatorialis)* and human dwellings [10]. *Rhodnius ecuadoriensis* is geographically separated from *R. pallescens* and *R. colombiensis* by the Andean mountains and by the pluvial forests of the Colombian Pacific coast [11]. *Rhodnius pallescens* inhabits palms trees, such as *Attalea butyracea* and *Cocos nucifera,* and is widely distributed across Central America and Colombia [11] in different climatic conditions and ecological zones [12], [13]. The most recently described species *R. colombiensis* [14] is completely a sylvatic species, and it is restricted to the inter Andean valley of Magdalena River in central Colombia. Although both *R. colombiensis* and *R. pallescens* species have been found in the same eco-geographical region of central Andean valleys in Colombia as well as inhabiting the same palm tree species (*A. butyracea)* [15], natural hybrids have not been reported. Due to the lack of knowledge concerning the inter-fertility between these sympatric species, chromosomal analyses of hybrids obtained from experimental crosses could help to identify its reproductive limits.

The genetic diversity within the Pacific group across its geographic distribution is only beginning to be ascertained, and apparent contradictory phylogenetic relationships among its members [16]–[18] suggest that further systematic studies are still needed.

Intraspecific differences of *R. pallescens* were accessed by cytogenetic, wing morphometric and molecular analysis of partial cytochrome b (cyt b) gene [19], [20]. Two divergent lineages are differentially distributed in Colombia (termed as *R. pallescens* I) and Central America (termed as *R. pallescens* II) as a consequence of both evolutionary diversification and environmental influence [19]. In *R. ecuadoriensis*, the analysis of mitochondrial cytochrome b gene [10], [21] and in the chromosome location of 45S rDNA cluster [22] suggested that Peruvian and Ecuadorian insects represent discrete populations or even incipient species [10], [21].

Although the Pacific group is unquestionably a monophyletic clade, several genetic incongruences among its species were detected by isoenzyme profiles [7] and nucleotide sequences comparisons of mitochondrial genes [16]–[18].

*Rhodnius colombiensis* was initially described as a sylvatic *R. prolixus* form occupying palms trees in central Andean valleys region of Colombia [7]. Its taxonomic status as a separate species was confirmed by isoenzyme and molecular analyses [14], [23]. The first assessment in phylogenetic reconstruction of Rhodniini tribe including the Pacific group`s species was performed by molecular and morphometric studies [7]. Isoenzymatic analysis of twelve enzyme systems as well as heads and wing measurements was performed in 13 *R. ecuadoriensis* specimens, 11 *R. pallescens* and 8 *R. colombiensis* (thought as “sylvatic *R. prolixus*” in that time) [7]. Cladograms inferred from isoenzyme alleles synapomorphies as well as in Mahalanobis distances derived from morphological measurements were congruent supporting the Pacific clade, but in this case a basal branch was observed for *R. pallescens* whereas *R. ecuadoriensis* and *R. colombiensis* were grouped in a derivate clade [7]. However, later phylogenetic and phylogeographic analyses using several mitochondrial genes (i.e. cytochrome b – cyt b or the large subunit ribosomal RNA - 16S) showed distinct phylogenetic arrays. A basal *R. pallescens* branch and derivate *R. ecuadoriensis - R. colombiensis* clade was observed in phylogenetic analysis based on cyt b gene [16], whereas basal *R. ecuadoriensis* branch and derivate *R. pallescens - R. colombiensis* clade was observed in phylogeographic analyses using 16S gene [17], [18]. Besides of the little information about chromosomal and genetic attributes of *R. colombiensis,* its phylogenetic relationship with *R. ecuadoriensis* and with recently identified *R. pallescens* lineages, as well as a credible hypothesis about its origin or evolutionary divergence with other Pacific species remained unresolved. Therefore further genetic analyses as well as new evolutionary hypotheses about Pacific group were still needed to understand the evolutionary trends that shape the phylogeographic and evolutionary landscape of *Rhodnius* Pacific group.

So far at least two theories about Pacific group origin (including *R. colombiensis* origin) have been proposed [8], [9]. Although both indicate a monophyletic origin from a widespread generalist species similar to *R. pictipes*, the first theory [8] suggests northwestern origin of the Pacific group, whereas the second [9] a southeastern origin.

In the northern origin theory is thought a basal *R. pallescens* clade was dispersed from northern Colombia to Central American, and across of the Caribbean coast and Andean valleys of Colombia, to eastern Ecuador and northern Peru giving thus origin to *R. pallescens* lineages, *R. ecuadoriensis* and *R. colombiensis* [8].

The second, and more recent theory about the evolution of the *Rhodnius* Pacific group suggests it may have occurred by a combination of adaptive radiation and vicariant processes [9], [10], [18]. This group is considered a lineage that derived from an ancestral population close to *R. pictipes* that reached the western side of the Andes range from the eastern Orinoco plains during the late Miocene (∼6 Mya) [10]. The rise of the Andean mountains during the Pliocene (∼5 Mya) split that population into two main clades: the “Colombian cluster”, comprising the ancestral forms of *R. pallescens*, and *R. colombiensis*. *Rhodnius* ecuadoriensis originated from an isolated pocket in the south, which adapted to new ecotopes [9].

In order to describe several chromosomal, genetic and morphological attributes of the poorly studied species *R. colombiensis* as well as clarify the phylogenetic picture and the species limits within the Pacific group, we performed cytogenetic, morphometric and phylogeographic studies based on the analysis of mitochondrial cyt b and ND4 genes and nuclear 28S gene of individuals belonging to *R. pallescens* lineages, *R. ecuadoriensis* and *R. colombiensis* taxa. Our findings provide new insights into the underlying biological processes that shape the evolution of this important group and improve the systematic picture within the genus *Rhodnius*.

## Materials and Methods

### Samples, DNA Extraction and PCR-amplification

**Figure 1:**
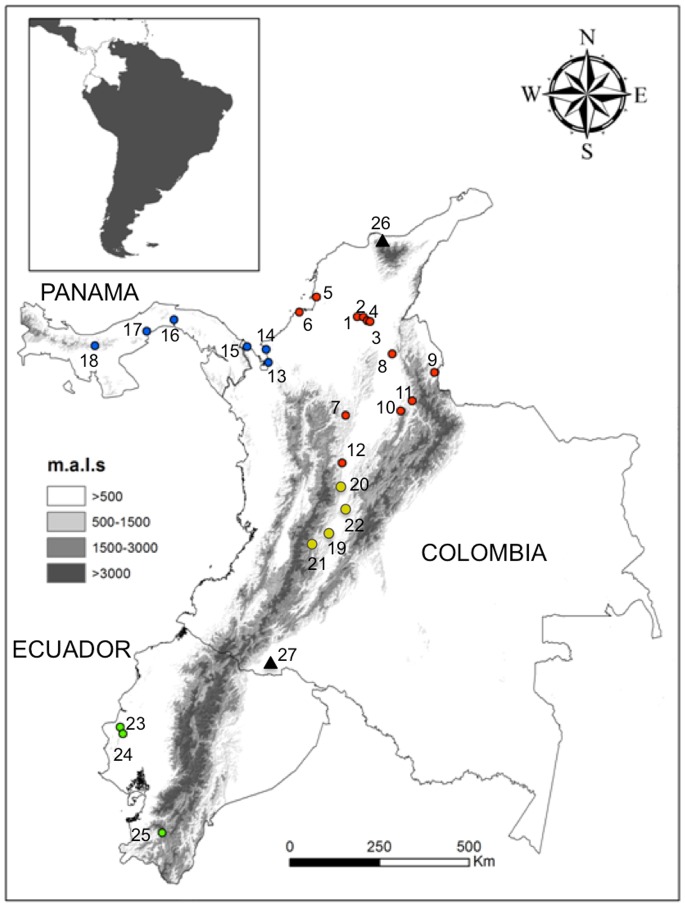
Collection sites of *Rhodnius* species of the Pacific group. Numbers indicated origin sites detailed in Table 1. Color indicates the species/lineages of the Pacific group: *R. pallescens* I in red; *R. pallescens* II in blue; *R. colombiensis* in yellow; and *R. ecuadoriensis* in green. Outgroup species *R. robustus* and *R*. *pictipes-*like are represented in black triangles. Numbers match with locality numbers in Table 1.

No specific permissions were required for insect collections performed in this work, and did not involve endangered or protected species. A total of 67 specimens representing 18 locations of *R. pallescens* from Colombia and Panama, 21 individuals of *R. colombiensis* from 4 locations, and 8 specimens of *R. ecuadoriensis* from 3 locations of Ecuador were included in our analyses (Table 1, Figure 1). Individuals were collected between 1997–2013. *R. pallescens* and *R. ecuadoriensis* identification was performed according to the morphological keys proposed by Lent and Wygodzinsky [24], and keys proposed for *R. colombiensis* by Moreno et al., [14].

**Table 1:** Geographic origins and number of specimens of the Pacific group used in this study.

Species/Lineage	Locality, country (number in Figure 1)	Code	ND4	cyt b	D2-28S	Morphometry*
*R. pallescens* I	San Zenón, Colombia (1)	RpalSze	2	2	2	0
	San Sebastián de Buenavista, Colombia (2)	RpalSsb	5	6 (3*)	5	20
	Mompós, Colombia (3)	RpalMom	3	4 *	4	3
	San Fernando, Colombia (4)	RpalSfe	1	1 *	1	0
	San Onofre, Colombia (5)	RpalSon	2	2	0	2
	San Bernando del Viento, Colombia (6)	RpalSba	1	1 *	1	4
	Vegachí, Colombia (7)	RpalVeg	4	5 (4*)	4	5
	Aguachica, Colombia (8)	RpalAgu	4	4 *	3	0
	El Carmen, Colombia (9)	RpalElc	3	3 (2*)	3	0
	San Vicente de Chucurí, Colombia (10)	RpalSvi	5	5 (3*)	5	0
	Bucaramanga, Colombia (11)	RpalBug	2	2 *	2	0
	Norcasía, Colombia (12)	RpalNor	3	4 *	2	15
*R. pallescens* II	Necoclí, Colombia (13)	RpalNec	4	6 (3*)	4	10
	Turbo, Colombia (14)	RpalTur	4	4 (2*)	1	0
	Acandí, Colombia (15)	RpalAca	5	5 *	5	0
	Chepo, Panamá (16)	RpalChe	4	4 *	4	0
	La Chorrera, Panamá (17)	RpalCho	4	3 *	4	0
	Santa Fe, Panamá (18)	RpalSF	4	6 (5*)	3	31
*R. colombiensis*	Coyaima, Colombia (19)	RcolTol	4	4	2	13
	Libano, Colombia (20)	RcolLib	5	4	4	0
	Chaparral, Colombia (21)	RcolCha	6	5	5	0
	Coello, Colombia (22)	RcolCoe	6	6	5	0
*R. ecuadoriensis*	Santa Ana, Ecuador (23)	RecuSA	2	2	2	0
	Portoviejo, Ecuador (24)	RecuPor	4	4	4	0
	Quilonga, Ecuador (25)	RecuQui	2	2	0	0
*R. pictipes-like*	Sierra Nevada de Santa Marta, Colombia (26)	RpicSNSM	2	2	0	0
*R. robustus-like*	Puerto Asís, Colombia (27)	RrobPut	2	1	0	0
TOTAL			93	97	76	103

* Number of specimens of *R. pallescens* previously analyzed in Gómez-Palacio et al., 2012.

Genomic DNA was obtained from leg or thorax muscles [25]. For each specimen a 631-bp fragment of nicotinamide adenine dinucleotide dehydrogenase 4 (ND4) gene was PCR-amplified using primers ND4-F (5′-TCAACATGAGCCCTTGGAAG -3′) and ND4-R (5′-TAATTCGTTGTCATGGTAATG -3′) [26]; and a 682-bp fragment of cytochrome B (cyt b) gene was PCR-amplified using primers CYTB7432 (5′-GGACGWGGWATTTATTATGGATC-3′) and CYTB7433 (5′-GCWCCAATTCARGTTARTAA-3′) [27]. PCR reactions for both mitochondrial genes were conducted in a final volume of 35 µl using 30-ng of DNA templates, 1X PCR buffer (0.1 M Tris–HCl, 0.5 M KCl, and 0.015 M MgCl2, pH 8.3), 250- µM dNTP, 0.016-µM of each primer, 35-mM MgCl2 and 2 U of Taq DNA polymerase. The fragments were amplified with the following thermal cycling conditions: 95°C for 5 min; 35 cycles of 94°C for 30 s, 50°C for 30 s, and 72°C for 60 s; 72°C for 10 min.

For the D2 variable region of the 28S rDNA gene (D2-28S) a fragment of 434-bp was PCR-amplified using primers D2F (5′-GCGAGTCGTGTTGCTTGATAGTGCAG-3′) and D2R, (5′-TTGGTCCGTGTTTCAAGACGGG-3′) [28]. PCR reactions were conducted in a final volume of 35 µl using 30-ng of DNA templates, 1X PCR buffer (0.1 M Tris–HCl, 0.5 M KCl, and 0.015 M MgCl2, pH 8.3), 250- µM dNTP, 0.025-µM of each primer, 3-mM MgCl2 and 2 U of Taq DNA polymerase (Promega®). After an initial denaturation of 95°C for 5 min, PCR reactions were 35 cycles at 95°C for 30 s, 60°C for 30 s, and 72°C for 30 s, followed by a final extension of 72°C for 7 min [29]. Amplicons were sent to Macrogen Inc., Korea to be purified and sequenced in both directions.

### Nucleotide Sequence Alignment and Diversity

All sequences used here are available in GenBank (accession numbers KC543506–66, and GQ850481, FJ229357–60, JQ686674–89 for those reported in Gómez-Palacio et al., (2012)). Alignments were made using CLUSTALW algorithm [30] implemented in Bioedit 7.0.5 [31]. ND4 and cyt b nucleotide sequences from specimens *R. pictipes-*like (form resembling *R. pictipes;* NJO, unpublished) collected in the Sierra Nevada de Santa Marta (SNSM) mountain in northern Colombia were also included in analyses. Colombian southern form of *R. robustus* [32] was included as outgroup in phylogenetic analyses (see below).

As a measure of gene diversity within the Pacific group, several parameters such as haplotype number (h), haplotype diversity (Hd), and nucleotide diversity (π) were calculated within species/lineages by using DNAsp 5.10.01 software [33]. Genetic distances among species/lineages were estimated for D2-28S, ND4 and cyt b genes based on the substitution model of K 2-p [34] as reported for *Rhodnius* sibling species [35], using MEGA 5.05 software [36].

### Phylogenetic Analysis

The combined data set of ND4 and cyt b (1313-bp) nucleotide sequences as well as the D2-28S fragment (434-bp) were used to infer the partitioned best-fitting model using the Akaike information criterion [37], as implemented in jMODELTEST 0.1.1 [38]. A Bayesian Metropolis coupling Markov Chain Monte Carlo (MC^3^) approach was implemented in BEAST 1.7.4 package [39]. Model parameters (base frequencies, transition/transversion ratio, rate variation shape parameter) were derived empirically, and two chains run for 10×10^9^ generations, with a sampling frequency of 10,000 generations. Finally by using as criteria the maximum sum of posterior probabilities (Maximum clade credibility) a final topology was chosen after discarding a burn-in of 10%.

Maximum Parsimony (MP) trees were constructed using TNT 1.1 [40]; shortest trees were found via a traditional heuristic search with tree bisection-reconnection (TBR) branch swapping saving 10 trees per replication, replacing existing trees. Statistical support for clades in the phylogenetic tree was assessed by the standard bootstrap method [41] with 100,000 replicates. Topologies were edited with the software FigTree 1.3.1. [42].

### Divergence Time Estimation

Based-tree topology a Maximum Likelihood (ML) test for molecular clock (it means that all tips of the tree are equidistant from the root of the tree) was performed using MEGA 5.05 [36] giving as result the rejection of a strict molecular clock model for the dataset. Thus a relaxed molecular clock model, which allows branch lengths to vary following an uncorrelated log-normal distribution [43] was performed using as prior assumption the independence of the clock models for both ND4 and cyt b data set using BEAST 1.7.4 package [39]. We considered a range of 15±5 Mya (before to the first rise of the Andes, dated about 10±7 Mya; [44]) as a calibration point for separation of trans-Andean *Rhodnius* species as inferred from formation of the Pebas System, a great Amazonian wetland that occurred in early and middle Miocene [44].

### Haplotype Network

Median-Joining haplotype network [45] of D2-28S fragment as well as combined ND4 and cyt b genes was performed to examine inter-haplotype relationships among species/lineages using Network 4.6.0.0 (http://www.fluxus-engineering.com). Haplotype networks were built using default parameters (equal character weight = 10; epsilon value = 10; transversions/transitions weight = 1∶1 and connection cost as a criterion).

### Total DNA Content Measured by Flow Cytometry in *R. colombiensis*

We measured DNA content only for *R. colombiensis* because *R. ecuadoriensis* and *R. pallescens* genome sizes were already known [19], [46]. The total nuclear DNA content of 13 specimens of *R. colombiensis* came from the type locality (Coyaima-Tolima) was measured from gonadal cells of male specimens as previously reported [47]. The cell DNA content was measured on an EPICS XL-MCL flow cytometer (Coulter Electronics, Hialeah, FL, USA) with an air-cooled argon-ion laser set at 488 nm and 15 mW. Propidium fluorescence (FL3), proportional to DNA content, was collected through a 650-nm DL dichroic filter fitted with a 625-nm BP band-pass filter. The DNA content in single cells was determined from FL3 linear histograms. For each sample, information for a minimum of 10,000 nuclear events was acquired using the System II software program (Beckman Coulter Inc., Brea, CA, USA). To evaluate the DNA content in picograms of DNA, a sample of normal human lymphocytes was fixed in ethanol/acetic acid and used as the standard reference (2C = 7.0 pg of DNA according to the Animal Genome Size Database (http://www.genomesize.com/). The absolute DNA amount was calculated with the ratio of the mean channel of the insect haploid peak to the mean channel of the human lymphocyte diploid G0/G1 peak.

### Experimental Crosses between *Rhodnius pallescens* and *R. colombiensis*, and Cytogenetic Analyses of F1 Progeny

Experimental crosses were performed between *R. colombiensis* and *R. pallescens* I (Colombian lineage). Individuals of *R. colombiensis* came from the type locality (Coyaima-Tolima) and individuals of *R. pallescens* lineage I were from Colombia (San Onofre-Sucre).

Crosses were made using three couples in each direction (male *R colombiensis* X female *R. pallescens*, or female *R. colombiensis* X male *R. pallescens*). All bugs used for the crosses were collected as nymphs; recently emerged (virgin) adults were place together in plastic vials with folded filter paper.

Testes from 6 freshly killed male hybrids (F1) were fixed in an ethanol–acetic acid mixture (3∶1) and stored at −20°C. Chromosome preparations and C-banding technique were performed as previously reported [48]. Females were not studied because meiotic stages are not usually observed in their ovaries.

### Geometric Morphometric Analysis

We identified morphological differences between *R. pallescens* lineages and *R. colombiensis* by performing two analyses, one based on wing size and the other for wing shape. Right wings were dissected and mounted using standard techniques as reported elsewhere [19] and photographed with a Nikon 990 digital camera fitted to a Nikon SMS 800 stereomicroscope. The wings were placed in the center of the visual field to reduce the risk of optical distortion. Ten landmarks were selected and the geometric coordinates of each landmark were digitalized using tpsDIG 2.16 [49].

To compare wing size among species/lineages, the “centroid-size” was used as isometric estimator of overall size derived from coordinate data [50]. It is defined as the square root of the sum of squared distances between the center of the configuration of landmarks and each individual landmark [50]. Differences in size were tested by one-way ANOVA and a post-hoc pairwise comparison was performed based on Tukey's HSD (honestly significant difference) test.

The Generalized Procrustes Analysis (GPA) superimposition algorithm implemented in the tpsRelw 1.11 [51] was used to obtain the wing shape variables. Shape variation was analyzed using the principal components of shape variables or relative warps. Differences in shape were tested by MANOVA. A post-hoc analysis by a pairwise Hotelling test (with Bonferroni correction) was performed when the MANOVA showed a significant overall difference between groups. A principal component analysis was performed to produce a scatter plot of specimens along the first two component axes, producing maximal and second to maximal separation between all groups. Additionally, a discriminant analysis (DA) reclassification test based on the first two discriminant functions (DF) to each observation was designed and the Mahalanobis distances among the shape’s centroids for each pair species/lineage (see Table 1) were compared. Statistics were performed using the free software PAST 1.94b [52], and the statistical significance of pairwise comparisons was tested by a null model using 1000 permutations.

## Results

### Sequence Variation and Genetic Distances

In the complete dataset a total of 121, 150 and 12 variable sites (S) were observed for ND4, cyt b and D2-28S genes, respectively, as well as 35, 33 and 8 haplotypes (h). Congruent gene diversity values for both ND4 and cyt b genes were detected within species/lineage Pacific group samples (Table 2). Nucleotide (π) diversity showed the lowest values in *R. colombiensis* (π = 0.004 for both ND4 and cyt b) and the highest within *R. pallescens* (π = 0.012 and 0.015), followed by *R. ecuadoriensis* (π = 0.011 and 0.009 for ND4 and cyt b, respectively). Haplotype diversity (Hd) was marginally similar among samples studied (Table 2). For D2-28S gene, 12 variable sites out of the 434-bp were observed across species/lineages, harboring the eight haplotypes (Table S1). Only one haplotype was species-specific to *R. colombiensis*, while the most frequent D2-28S haplotype was shared for both *R. pallescens* lineages and *R. ecuadoriensis*, but not by *R. colombiensis* (Table S1).

**Table 2:** Summary of genetic diversity indices Notations: h: number of haplotypes; Hd (± SD): haplotype diversity (standard deviation); π (± SD): nucleotide diversity (standard deviation).

Species/lineage	Mitochondrial gene					
	ND4			cyt b		
	h	Hd (± SD)	π (± SD)	h	Hd (± SD)	π (± SD)
*R. pallescens I*	19	0.931 (0.030)	0.012 (0.002)	18	0.861 (0.049)	0.012 (0.001)
*R. pallescens II*	7	0.667 (0.094)	0.010 (0.002)	6	0.794 (0.037)	0.015 (0.003)
*R. colombiensis*	4	0.762 (0.048)	0.004 (4×10^−4^)	5	0.788 (0.055)	0.004 (5×10^−4^)
*R. ecuadoriensis*	5	0.893 (0.086)	0.011 (0.002)	4	0.857 (0.082)	0.009 (0.001)

K 2-p-based genetic distances (Table 3) were moderately high between *R. pallescens* lineages (d = 0.041 and 0.051 for ND4 and cyt b genes respectively). Similar values were observed between *R. pallescens* I vs. *R. colombiensis* (d = 0.034 and 0.065 for ND4 and cyt b, respectively), and between *R. pallescens* II vs. *R. colombiensis* (d = 0.045 and 0.062 for ND4 and cyt b, respectively). The highest genetic distance was estimated between *R. pallescens* II and *R. ecuadoriensis* (d = 0.102 and 0.133 for ND4 and cyt b, respectively). For the D2-28S gene, estimated K 2-p-based genetic distances were remarkably low between *R. pallescens* lineages (d = 0.001), and between those with *R. ecuadoriensis* (d = 0.001, for both comparisons); but comparatively higher when *R. colombiensis* was included (*R. pallescens* I vs. *R. colombiensis* d = 0.005; *R. pallescens* II vs. *R. colombiensis* d = 0.006; and *R. ecuadoriensis* vs. *R. colombiensis* d = 0.005).

**Table 3:** Pairwise K 2-p-based genetic distance (upper right) and cyt b (lower left) gene fragments.

	*R.* *pallescens* I	*R.* *pallescens* II	*R.* *colombiensis*	*R.* *ecuadoriensis*
*R. pallescens* I		0.041	0.034	0.095
*R. pallescens* II	0.057		0.045	0.102
*R. colombiensis*	0.065	0.062		0.089
*R. ecuadoriensis*	0.133	0.133	0.128	

### Phylogenetic Results and Divergence

**Figure 2:**
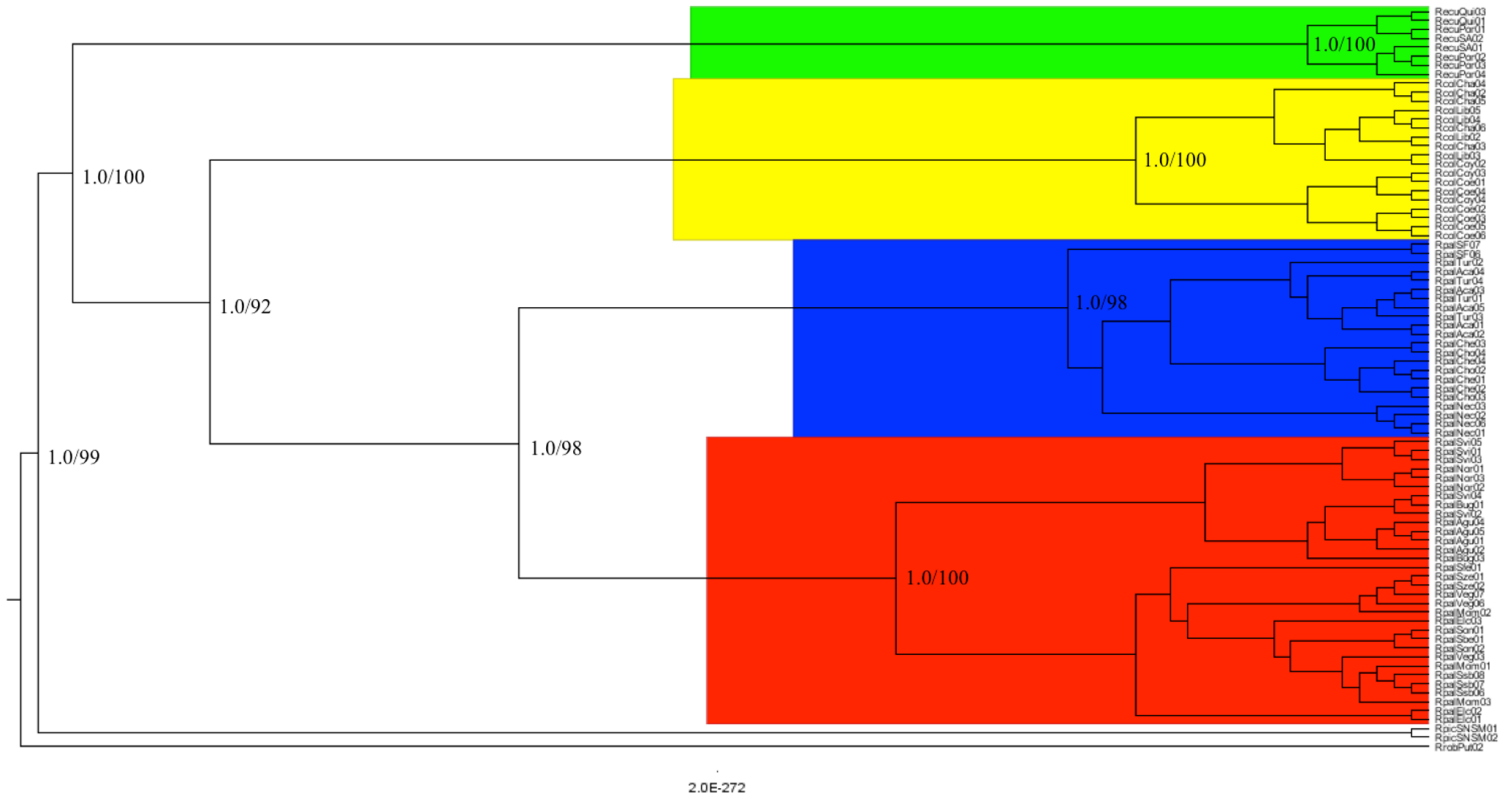
Bayesian consensus tree for the combined ND4 and cyt b genes. Posterior probabilities/bootstrap support (from MP analysis, Figure S1) for representative nodes is shown. Color of clades indicate the Pacific group species/lineages: *R. pallescens* I in red; *R. pallescens* II in blue; *R. colombiensis* in yellow; and *R. ecuadoriensis* in green.

Due to a low number of variable sites observed in D2-28S gene, no resolute topologies were obtained for this marker (data not shown). For the combined ND4 and cyt b dataset partitioned substitution GTR+G and HKY+I models were used respectively. MP strict consensus tree showed 673 steps, with a consistency index of 0.74 and a retention index of 0.95 (Figure S1). Both Bayesian and MP analyses showed identical topologies; hence only the Bayesian tree is shown (Figure 2). Four well-supported monophyletic clades (posterior probabilities = 1.0, and bootstrap values >0.9) were observed harboring the three species and both *R. pallescens* lineages (Figure 2). Basal clade was composed of *R. ecuadoriensis* followed by a clade belonging to *R. colombiensis,* and both lineages of *R. pallescens* (Figure 2). Based on a relaxed clock model Bayesian analysis indicated that *R. ecuadoriensis* clade diverged from the ancestral Pacific group at 11.5 (±3.9 Mya); followed by the divergence of *R. colombiensis* (10±3.4 Mya); and *R. pallescens* lineages (6.1±2.1 Mya).

### mtDNA and rDNA Haplotype Network

**Figure 3:**
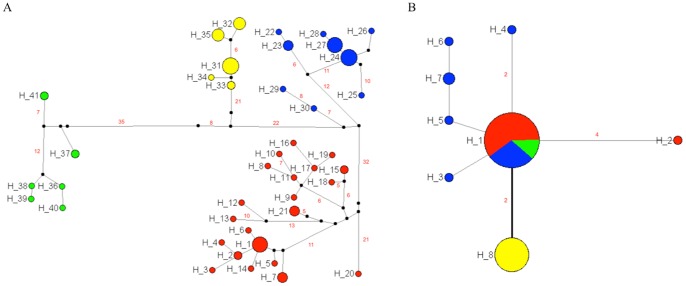
Haplotype network of (A) combined ND4 - cyt b genes, and (B) D2-28S gene of the Pacific group species/lineages. The size of the circles indicates the haplotype frequency. Mutation number for every node (>5 for mtDNA and >1 for D2-28S gene) are detailed in (A) and (B) in red. Color of the pie indicates the species/lineage of the Pacific group: *R. pallescens* I in red; *R. pallescens* II in blue; *R. colombiensis* in yellow; and *R. ecuadoriensis* in green. Line in bold in (B) shows two mutation steps separating *R. colombiensis* from the central haplotype.

The haplotype network based on 41 haplotypes of the combined ND4 and cyt b dataset (Table S2) shows four clearly separated groups belonging to the three species, plus the two *R. pallescens* lineages (Figure 3A). Moreover for D2-28S gene a star-shape haplotype network was observed (Figure 3B). The most frequent D2-28S haplotype included all *R. pallescens* I and *R. ecuadoriensis* individuals (32 and 6 respectively), and most *R. pallescens* II specimens (15 individuals) (Figure. 3B and Table S1). From this haplotype derived the remaining haplotypes of *R. pallescens* II and *R. colombiensis* (Figure 3B). This last species differs by two mutational steps from the principal haplotype (Figure 3B).

### Genome Size of *R. colombiensis*

Based on DNA flow cytometric profiles obtained (Figure S2), genome size measure in *R. colombiensis* indicated an amount of haploid DNA content (C value) of 0.58±0.01 pg.

### Experimental Crosses between *Rhodnius pallescens* and *R. colombiensis*, and Cytogenetic Analyses of F1 Progeny

Crosses between *R. pallescens* lineage I (males) and *R. colombiensis* (females) resulted in a F1 progeny that include both adult males and females. In the reciprocal cross *R. pallescens* lineage I females and *R. colombiensis* males, the eggs did not eclose and thus no progeny was obtained.

**Figure 4:**
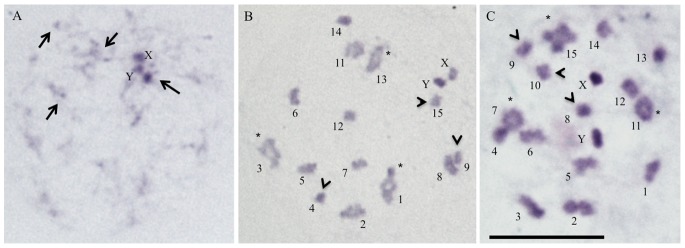
Male meiosis of hybrids (F1 progeny) from experimental crosses by C-banding technique. The crosses were made between *R. pallescens* (males) and R. *colombiensis* (females). (A) Early meiotic prophase (diffuse stage) showing a heteropycnotic chromocenter constituted by XY sex chromosomes plus one autosomal C-heterochromatic dot. Other C-dots dispersed in the nucleus (arrows) are observed. (B) Late diplotene or diakinesis and (C) Metaphase I showing a variable number of chromosomes, instead of twelve (ten autosomal pairs plus 2 sex chromosomes) observed in normal cells. Some of autosomes are bivalents (asterisks) and other are univalents (arrowheads).

All male hybrids had the same diploid chromosome number as the progenitors (2n = 22) consisting of 20 autosomes and a pair of sex chromosomes (XY). Mitotic chromosomes have C-blocks or were completely euchromatic (Figure 4). Chromosomes were rather similar in size and it was not possible to establish if they came from *R. colombiensis* or *R. pallescens*. Early meiotic cells appeared normal and had a heteropycnotic chromocenter constituted by the associated XY sex chromosomes plus autosomal heterochromatin (Figure 4a). Later meiotic stages had several meiotic anomalies that affected spermatogenesis to varying degrees. Chromosomal pairing was greatly altered during first meiotic division. Some autosomes synapsed and formed bivalents, while others remained as univalents (Figures 4b and 4c). The frequencies of bivalents and univalents varied among different individuals and between cells of the same specimen. The number of cells that progress through meiosis was drastically reduced and the resulting cells are expected to have unbalanced genomes as a consequence of missegregation during first meiotic division.

### Wing Size and Shape Comparison among *R. pallescens* Lineages and *R. colombiensis*

**Figure 5:**
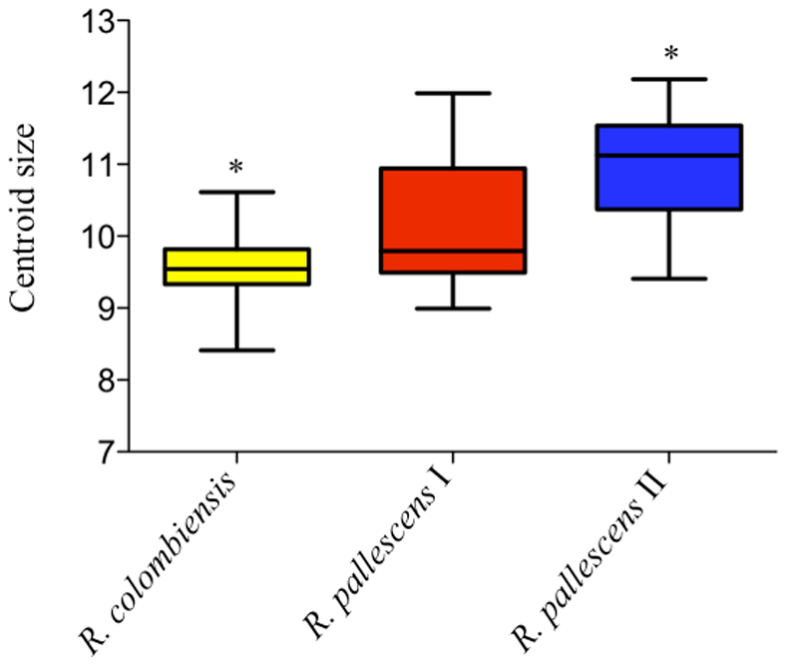
Wing size differences of *R. colombiensis* and *R. pallescens*. There were used specimens of the two Colombian-Central American *R. pallescens* lineages (termed as *R. pallescens* I and II respectively). The arithmetic median can be observed as a line that divides the boxes into two. The ends of the boxes correspond to the 25% and 75% quantiles; the vertical lines show the maximum and minimum value of the centroid size distribution. * Indicate significant differences (p<0.001).

Significant differences in size were detected among all species/lineages (F = 24.70, p<0.001). After a Tukey HSD post-hoc test, no significant differences (p>0.01) in size were identified between the smallest insects *R. colombiensis* and *R. pallescens* I, but both of them were significant differentiated (p<0.01) from the largest ones *R. pallescens* II (Figure 5 and Table 4).

**Table 4:** ANOVA and Tukey HSD test of pairwise comparisons.

ANOVA						
Variation source	Sum of sqrs	df	Mean square	F		p-value
Among species/lineages	27.08	2	13.54	24.7		1.00E−05
Within species/lineages	55.35	101	0.5481			
Total	82.43	103				
**Tukey's HSD (Honestly Significant Difference) test**						
	**Mean differences**	**q**	**Significant (p<0.01)?**		**95% CI of differences**	
*R. pallescens* I vs *R. pallescens* II	−0.8665	7.857	Yes		−1.333 to −0.4004	
*R. pallescens* I vs *R. colombiensis*	0.5548	3.404	No		−0.1339 to 1.244	
*R. pallescens* II vs *R. colombiensis*	1.421	8.53	Yes		0.7172 to 2.126	

The tests were based on wing size for *R. colombiensis*, and Colombian-Central American *R. pallescens* lineages (termed as *R. pallescens* I and II respectively).

**Figure 6:**
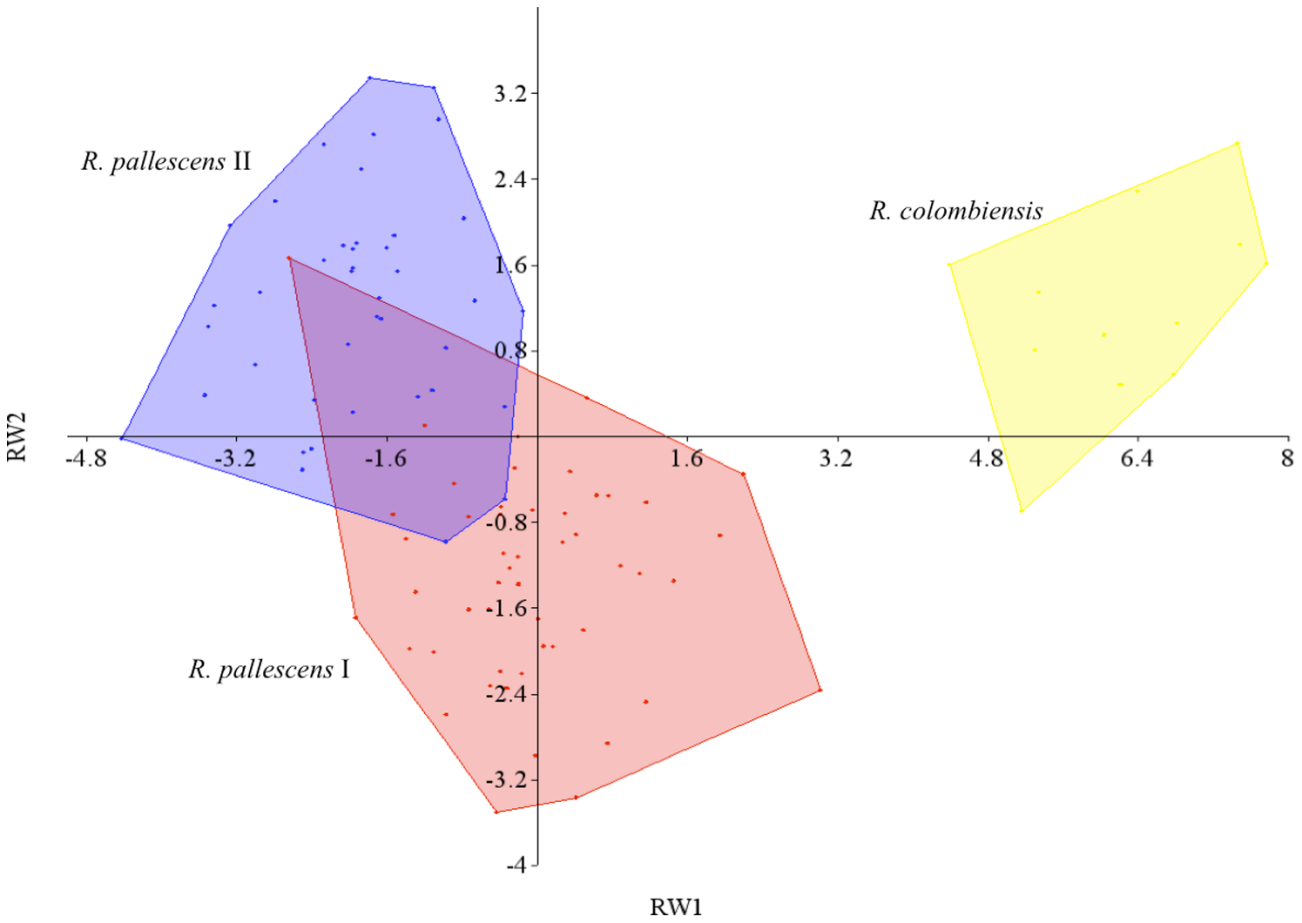
Morphometric analysis of male wings in *R. pallescens* lineages and *R. colombiensis*. The polygons represent the shape of wings projected on the first (x-axis) and second relative warp (y-axis), which were derived from a relative warp analysis. RW1 explains 40.6% of the variance while RW2 explains 12.5%. For easy visualization of populations the lines connect the most external individuals and filled color represents species/lineage: *R. colombiensis* in yellow; *R. pallescens* I in red and *R. pallescens* II in blue.

The first two axes of relative warps explained 40.6% and 12.5% of the variation, respectively, corresponding to 53.1% of the total variation of the specimens’ wing shape, dismissing the use of other axes, and shape differences were detected between *R. pallescens* lineages and *R. colombiensis* (Figure 6). The MANOVA test allowed detecting significant differences in wing shape among all species/lineages (F = 18.70, p<0.001) (Table 5). The Hotelling post-hoc analysis showed that significant differences in wing shape were detected between all pairwise species/lineages (Table 5). Reclassification of specimens based on discriminant factors resulted in the correct classification of 96.12% of the individuals to their attributed species/lineage. Reclassification was perfect for the individuals of *R. colombiensis* (100%), followed by *R. pallescens* I (97.9%), and *R. pallescens* II (92.7%) (Table 6).

**Table 5:** MANOVA and Hotelling pairwise comparisons based on shape variation.

Wilk's Lamda	df1	df2	F	p
0.0498	32	172	18.7	6.988 E−41
Hotelling's pairwise comparisons, Bonferroni corrected/Square Mahalanobis distances				
	*R. pallescens I*	*R. pallescens II*	*R. colombiensis*	
*R. pallescens* I	−	10.0471	45.9963	
*R. pallescens* II	**5.72E**−**14**	−	66.9548	
*R. colombiensis*	**5.43E**−**16**	**9.45E**−**16**	−	

Mahalanobis distances are given above the diagonal and Hotelling p-values with Bonferroni correction values are given below the diagonal. Bold values indicate significant differences (p<0.001).

**Table 6:** Reclassification analysis of individuals based on wing shape.

Species/lineage	Given group	Sell species/lineage	Other species/lineage	Clasification percent
*R. pallescens* I	49	48	1	97.9
*R. pallescens* II	41	38	3	92.7
*R. colombiensis*	13	13	0	100

Given group is the group assigned based on morphology/identification key, self and other species/lineage is group assigned based on discriminant factors of the wing shape analysis. Classification percent correspond to the percentage of individuals match between given and self group.

## Discussion

### Phylogenetic Trends in the Pacific Group Species

Phylogeny supports that *R. colombiensis* is a sister taxa of *R. pallescens* and that both have diverged from an ancestral clade that includes *R. ecuadoriensis*. We suggest additional timing for the Pacific group origin according to relaxed clock estimate divergence time by using as calibration point the formation of the Pebas wetland dated at middle Miocene [44], which is thought to have the most important role in *cis* and *trans-Andean* higher lineages diversification in Rhodniini. We estimate that *R. ecuadoriensis* and *R. colombiensis* divergence occurred in late Miocene (∼11 to 7 Mya), a period when the Northern Andes had reached no more than half of their modern elevation [53]; and the diversification of the *R. pallescens* lineages occurred during the latest Miocene-early Pliocene, being contemporary to the rapid uplift and extensive emergence of the Central American isthmus [54]. Genetic distances of ND4 and cyt b genes indicates that *R. ecuadoriensis* is the most divergent species, while similar values are evidenced between *R. pallescens* lineages, and between them with *R. colombiensis,* indicating that molecular differentiation between *R. pallescens* and *R. colombiensis* species is almost as recent as the arising two lineages with *R. pallescens*. This assumption was also supported by molecular relaxed clock results shown here.

Several genetic and phenotypic attributes of *R. ecuadoriensis, R. colombiensis* and *R. pallescens* explored here suggest that the Pacific group evolution involved at least two macro-evolutionary processes that gave rise to the current phylogeographic distribution: i) a wide geographical dispersion (from south to north) along the northern Andes during uplifting that separated *R. ecuadoriensis* and *R. colombiensis* basal populations, and ii) the formation of the Panamian isthmus that extended the spread of *R. pallescens*. These hypotheses support the southeastern origin theory of Pacific group that is suggested as a combination of adaptive radiation and vicariant processes [9], [10], [18].

### Taxonomic Validity of the Pacific Group Species

Specific status of *R. colombiensis* was initially proposed by isoenzymatic differentiation and by comparison of morphometric and genitalic structures [7], [14]. However, data about its evolutionary history and genetic relationship with its closer conspecific species *R. pallescens* were lacking. Here, we explored for the first time the biological differentiation of *R. colombiensis* within the Pacific group using molecular and morphometric analyses. Our results support the taxonomic status of *R. colombiensis* as a *R. pallescens* sibling species. Genetic distance estimates of mtDNA among Pacific group species (ranged between d = 0.034 to 0.133) were similar to those observed in *cis-Andean* species, specifically in cryptic species belonging to robustus lineage (that ranged between d = 0.023 to 0.072) [35]. The chromosomal and genome size measurements suggest relevant genomic arrangements could be involved in *R. colombiensis* speciation, suggesting distinct evolutionary trends were placed in the Pacific group evolution.

Chromosomal traits and genome size in Triatominae had been broadly used in understanding the taxonomy, diversification and genome evolution in several species of this subfamily [46]. The three species of Pacific group possess similar chromosomal characteristics, such as the same number of autosomes (20), sex mechanism (XY in males and XX in females) and the presence of several autosomal pairs with small terminal C-dots [20], [55]. For these reasons it is very difficult to differentiate these three species by standard cytogenetic analyses but the chromosomal data presented here suggested that striking structural chromosome rearrangements occurred during the divergence of these species. The haploid DNA content for *R. pallescens* was 0.73±0.04 pg [19], similar to that observed in *R. ecuadoriensis* (0.72 pg) [47]. Our results revealed that *R. colombiensis* has the lowest value of DNA content (0.58±0.01 pg) and suggest that DNA loss has taken placed during the evolution of this species. Recent analyses about the chromosomal location of ribosomal genes (45S rDNA clusters) also indicated a striking genome differentiation among these species. Fluorescent *in situ* hybridization assays shown that ribosomal rDNA clusters are located in both XY sex chromosomes in *R. pallescens* and in Ecuadorian populations of *R. ecuadoriensis,* while that they are located only in the X chromosome of *R. colombiensis* as well as in some specimens of the Peruvian *R. ecuadoriensis* populations [22], [55].

The mechanisms that limit gene flow between populations and species can be studied by performing experimental crosses and analyzing the progeny [56]. Hybridization studies have been implemented in several species of the *Triatoma* and *Meccus* genera (for review see [57]–[59]. The present study constitutes the first cytogenetic analysis of experimental hybrids among *Rhodnius* species. The analysis of the crosses between *R. colombiensis* and *R. pallescens* reveals the existence of both pre-zygotic and post-zygotic reproductive barriers. The crosses *R. pallescens* lineage I (females) and *R. colombiensis* (males) did not produce progeny, indicating that non-fertile eggs were obtained by chance or supporting the possible existence of a pre-zygotic isolation mechanism (e.g. incompatibilities between genitalia of both species). Crosses between *R. pallescens* lineage I (males) and *R. colombiensis* (females) overcome pre-zygotic barriers but produce infertile F1 hybrids (post-zygotic reproductive barrier). Sterility was associated with failures in chromosome pairing during meiosis (Figure 4) that led to the production of unbalanced gametes as observed in other interspecific triatomine hybrids [58], [59]. The observed isolation mechanisms explain the lack of natural hybrids although both species are sympatric in the inter Andean valley of Magdalena River and occupy the same sylvatic ecotope (*A. butyracea* palm trees).

The alteration in the meiotic pairing in the gonad cells of the hybrids between *R. pallescens* and *R. colombiensis* also reveals the lack of genetic homology between chromosomes of both species. This result, together with the variation in genome size and rDNA chromosome location between the species [22], indicates that drastic genome rearrangements may have occurred between the species, giving rise to the speciation event in *R. colombiensis*.

Patterns of morphological variation that involves size or shape dimensions have been often interpreted with regard to their evolutionary importance [60]. Environmental influences (such as elevation and humidity) are well known in several Triatominae species (reviewed in [61]), and while wing size differentiation has been suggested to be strongly influenced (but not exclusively) by ecological attributes of species and populations, wing shape is thought often more affected by genetic and historical attributes of the evolutionary process. Under this consideration, as *R. pallescens* and *R. colombiensis* species are sympatric and have ecological similarity, they were expected to have similar wing sizes but large differences in wing shape are evident between them. These results unveil different biological (genetic) and ecological (environmental) pressures influencing morphological diversity within both species.

## Conclusions

In summary, we consider that different rates of molecular divergence detected in mtDNA and rDNA sequences; genome size variation and rDNA location in *R. colombiensis*, the evidence of post-zygotic barriers, and wing shape differentiation between sympatric species *R. pallescens* and *R. colombiensis*, indicate that distinct evolutionary trends are drivers of the evolutionary change within the Pacific group. Similar studies should be extended to determine the evolutionary history of the larger pictipes group.

## Supporting Information

Figure S1 Maximum Parsimony tree for the combined ND4 and cyt b genes. Bootstrap support for representative nodes is shown. Color of clades indicate specie/lineage: *R. pallescens* I in red; *R. pallescens* II in blue; *R. colombiensis* in yellow; and *R. ecuadoriensis* in green.
(TIF)

Figure S2 Representative DNA flow cytometric histogram showing the distribution of testis cells from *R. colombiensis*. Relative intensity (in arbitrary units) of DNA associated PI fluorescence are shown on the x-axis. The corresponding number of cells is displayed on the y-axis. (A) Human polimophonuclear leukocytes and (B) *R. colombiensis*. Mean of PI fluorescence of M1 peak indicating C-value is shown.
(TIF)

Table S1 Multiple alignment of D2-28S gene sequences of *Rhodnius* Pacific group species. Codes and origins are shown in Table 1. Identical bases are represented by a dot (.). Only variable sites, with sequence positions given above, are shown.
(DOC)

Table S2 Haplotype description of combined ND4 and cyt b genes for *Rhodnius* Pacific group species/lineages.
(DOC)
