# Supplementary figures and images for: Genetic, Cytogenetic and Morphological Trends in the Evolution of the Rhodnius (Triatominae: Rhodniini) Trans-Andean Group
Source: PLoS One. 2014 Feb 3;9(2):e87493. doi: 10.1371/journal.pone.0087493 (PMC3911991; doi:10.1371/journal.pone.0087493)

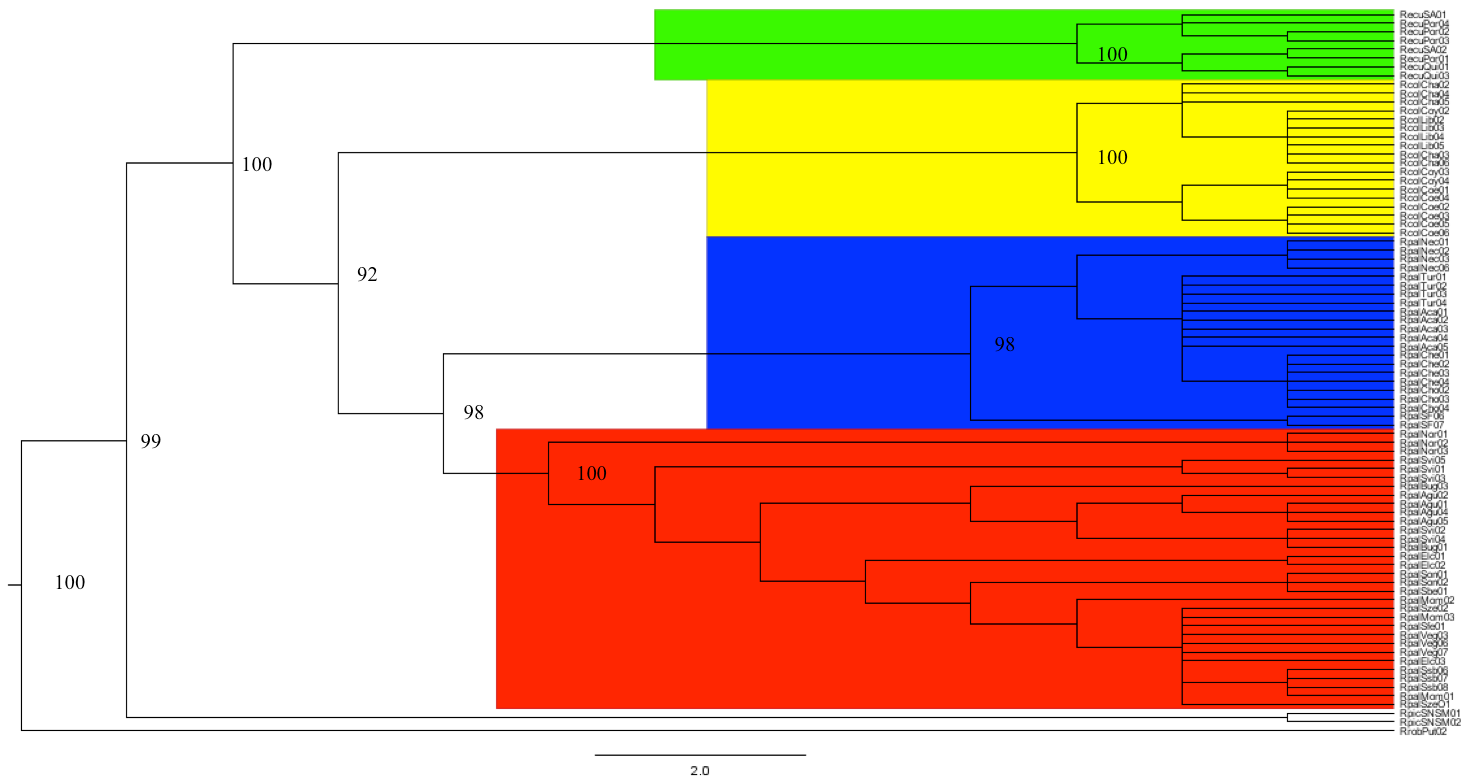

Supplement: Figure S1 — Maximum Parsimony tree for the combined ND4 and cyt b genes. Bootstrap support for representative nodes is shown. Color of clades indicate specie/lineage: R. pallescens I in red; R. pallescens II in blue; R. colombiensis in yellow; and R. ecuadoriensis in green. (TIF) [file pone.0087493.s001.tif]

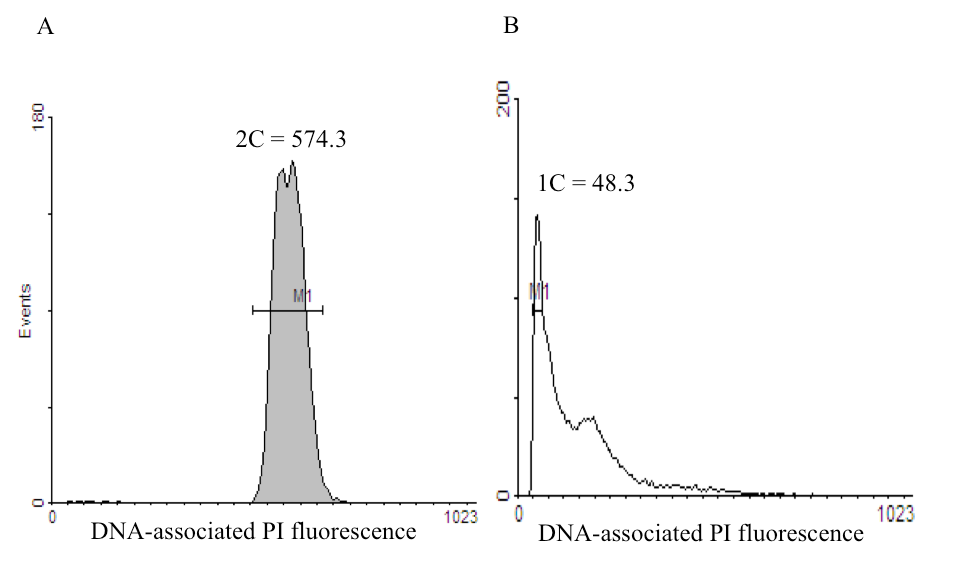

Supplement: Figure S2 — Representative DNA flow cytometric histogram showing the distribution of testis cells from R. colombiensis. Relative intensity (in arbitrary units) of DNA associated PI fluorescence are shown on the x-axis. The corresponding number of cells is displayed on the y-axis. (A) Human polimophonuclear leukocytes and (B) R. colombiensis. Mean of PI fluorescence of M1 peak indicating C-value is shown. (TIF) [file pone.0087493.s002.tif]
