# Supplementary material for: Genetic, Cytogenetic and Morphological Trends in the Evolution of the Rhodnius (Triatominae: Rhodniini) Trans-Andean Group
Source: PLoS One. 2014 Feb 3;9(2):e87493. doi: 10.1371/journal.pone.0087493 (PMC3911991; doi:10.1371/journal.pone.0087493)
Supplement: Table S1 — Multiple alignment of D2-28S gene sequences of Rhodnius Pacific group species. Codes and origins are shown in Table 1. Identical bases are represented by a dot (.). Only variable sites, with sequence positions given above, are shown. (DOC) [file pone.0087493.s003.doc]

**Supplementary Table S1**. Multiple alignment of D2-28S gene sequences of *Rhodnius* Pacific group species (*R. pallescens, R. colombiensis* and *R. ecuadoriensis)* studied. Codes and origins are shown in Table 1 and Figure 1. Colors of haplotypes are the same shown in Figure 3B. Identical bases are represented by a dot (.). Only variable sites with sequence positions given above are shown.

222333444

248036146013

205256329401

RpalSze01 CAAGTAACCTCG

RpalSze02 ............

RpalSsb01 ............

RpalSsb02 ............

RpalSsb06 ............

RpalSsb07 ............

RpalSsb08 ............

RpalSbe01 ............

RpalMom01 ............

RpalMom02 ............

RpalMom03 ............

RpalMom04 ............

RpalSfe01 ............

RpalVeg01 ............

H_1

RpalVeg03 ............

RpalVeg06 ............

RpalVeg07 ............

RpalAgu01 ............

RpalAgu04 ............

RpalAgu05 ............

RpalElc01 ............

RpalElc02 ............

RpalElc03 ............

RpalSvi01 ............

RpalSvi02 ............

RpalSvi03 ............

RpalSvi04 ............

RpalSvi05 ............

RpalBug01 ............

RpalBug03 ............

RpalNor01 ............

RpalNor02 ............

H_2

RpalNor03 ..C.C.CT....

RpalNec01 ............

H_1

RpalNec02 ............

RpalNec03 ............

H_3

RpalNec06 ............

RpalTur01 ........T...

RpalAca01 ............

RpalAca02 ............

RpalAca03 ............

H_1

RpalAca04 ............

RpalAca05 ............

RpalChe01 ............

H_4

RpalChe02 ............

RpalChe03 ...A.......A

H_1

RpalChe04 ............

H_5

RpalCho01 T...........

RpalCho02 ............

H_1

RpalCho03 ............

RpalCho04 ............

H_6

RpalSF01 TC...G......

H_7

RpalSF06 T....G......

RpalSF07 T....G......

RcolCoy02 .........AT.

RcolCoy03 .........AT.

RcolLib02 .........AT.

RcolLib03 .........AT.

RcolLib04 .........AT.

H_8

RcolLib05 .........AT.

RcolCha01 .........AT.

RcolCha02 .........AT.

RcolCha03 .........AT.

RcolCha05 .........AT.

RcolCha06 .........AT.

RcolCoe02 .........AT.

RcolCoe03 .........AT.

RcolCoe04 .........AT.

RcolCoe05 .........AT.

RecuSA01 ............

RecuSA02 ............

H_1

RecuPor01 ............

RecuPor02 ............

RecuPor03 ............

RecuPor04 ............
