# Supplementary material for: Genetic, Cytogenetic and Morphological Trends in the Evolution of the Rhodnius (Triatominae: Rhodniini) Trans-Andean Group
Source: PLoS One. 2014 Feb 3;9(2):e87493. doi: 10.1371/journal.pone.0087493 (PMC3911991; doi:10.1371/journal.pone.0087493)
Supplement: Table S2 — Haplotype description of combined ND4 and cyt b genes for Rhodnius Pacific group species/lineages. (DOC) [file pone.0087493.s004.doc]

# Supplementary Table S2. Haplotype description of combined ND4 and cyt b genes for *Rhodnius* Pacific group species/lineages. Samples description is shown in Table 1.

| **Species/lineage** | **Haplotype code** | **Frecuency** | **Samples** |
| --- | --- | --- | --- |
| *R. pallescens* I | Hap_1 | 6 | RpalSzeO1, RpalSze02, RpalMom03, RpalVeg03, RpalVeg06, RpalVeg07 |
|  | Hap_2 | 2 | RpalSsb06, RpalSsb07 |
|  | Hap_3 | 1 | RpalSsb08 |
|  | Hap_4 | 1 | RpalMom01 |
|  | Hap_5 | 1 | RpalMom02 |
|  | Hap_6 | 1 | RpalSfe01 |
|  | Hap_7 | 3 | RpalSon01, RpalSon02, RpalSbe01 |
|  | Hap_8 | 1 | RpalAgu01 |
|  | Hap_9 | 1 | RpalAgu02 |
|  | Hap_10 | 1 | RpalAgu04 |
|  | Hap_11 | 1 | RpalAgu05 |
|  | Hap_12 | 1 | RpalElc01 |
|  | Hap_13 | 1 | RpalElc02 |
|  | Hap_14 | 1 | RpalElc03 |
|  | Hap_15 | 2 | RpalSvi01, RpalSvi03 |
|  | Hap_16 | 1 | RpalSvi02 |
|  | Hap_17 | 1 | RpalSvi04 |
|  | Hap_18 | 1 | RpalSvi05 |
|  | Hap_19 | 1 | RpalBug01 |
|  | Hap_20 | 1 | RpalBug03 |
|  | Hap_21 | 3 | RpalNor01, RpalNor02, RpalNor03 |
| *R. pallescens* II | Hap_22 | 1 | RpalNec01 |
|  | Hap_23 | 3 | RpalNec02, RpalNec03, RpalNec06 |
|  | Hap_24 | 7 | RpalTur01, RpalTur03, RpalAca01, RpalAca02, RpalAca03, RpalAca04, RpalAca05 |
|  | Hap_25 | 1 | RpalTur02 |
|  | Hap_26 | 1 | RpalTur04 |
|  | Hap_27 | 6 | RpalChe01, RpalChe02, RpalChe03, RpalChe04, RpalCho02, RpalCho03 |
|  | Hap_28 | 1 | RpalCho04 |
|  | Hap_29 | 1 | RpalSF06 |
|  | Hap_30 | 1 | RpalSF07 |
| *R. colombiensis* | Hap_31 | 7 | RcolCoy02, RcolLib02, RcolLib03, RcolLib04, RcolLib05, RcolCha03, RcolCha06 |
|  | Hap_32 | 4 | RcolCoy03, RcolCoy04, RcolCoe01, RcolCoe04 |
|  | Hap_33 | 2 | RcolCha02, RcolCha05 |
|  | Hap_34 | 1 | RcolCha04 |
|  | Hap_35 | 4 | RcolCoe02, RcolCoe03, RcolCoe05, RcolCoe06 |
| *R. ecuadoriensis* | Hap_36 | 1 | RecuSA01 |
|  | Hap_37 | 2 | RecuSA02, RecuPor01 |
|  | Hap_38 | 1 | RecuPor02 |
|  | Hap_39 | 1 | RecuPor03 |
|  | Hap_40 | 1 | RecuPor04 |
|  | Hap_41 | 2 | RecuQui01, RecuQui03 |
